# Curdione induces ferroptosis mediated by m6A methylation via METTL14 and YTHDF2 in colorectal cancer

**DOI:** 10.1186/s13020-023-00820-x

**Published:** 2023-09-21

**Authors:** Fang Wang, Zheng Sun, Qunyao Zhang, Hao Yang, Gang Yang, Qi Yang, Yimiao Zhu, Wenya Wu, Wenwen Xu, Xiaoyu Wu

**Affiliations:** 1 First Clinical Medical College, Nanjing University of Chinese Medicine, Nanjing, 210046 Jiangsu China; 2 Department of Surgical Oncology, The Affiliated Hospital of Nanjing University of Chinese Medicine, Nanjing, 210029 Jiangsu China; 3 Department of Gynecology, The Affiliated Hospital of Nanjing University of Chinese Medicine, Nanjing, 210029 Jiangsu China

**Keywords:** Curdione, m6A, Colorectal cancer, Ferroptosis

## Abstract

**Background:**

Curdione is a sesquiterpene isolated from *Curcumae Rhizoma* that possesses high biological activity and extensive pharmacological effects. As a traditional Chinese medicine, *Curcumae Rhizoma* can inhibit the development of many types of cancer, especially colorectal cancer. However, the anti-colorectal mechanism of its monomer curdione remains unclear.

**Methods:**

Colorectal cancer (CRC) cells were treated with curdione at doses of 12.5 μM, 25 μM, and 50 μM, and then the cells’ activity was measured with methyl thiazolyl tetrazolium (MTT). Nude mice were administered different doses of curdione subcutaneously and oxaliplatin by tail vein injection, and then hematoxylin–eosin (HE) staining was adopted to examine tumor histology. Moreover, flow cytometry was applied to detect reactive oxygen species in cells and tissues. Kits were employed to detect the levels of iron ions, malondialdehyde, lipid hydroperoxide, and glutathione. Polymerase chain reaction (PCR) and Western blotting were adopted to detect ferroptosis and m6A modification-related factors. A methylation spot hybridization assay was performed to measure changes in overall methylation. SLC7A11 and HOXA13 were measured by MeRIP-qPCR. The shRNA-METTL14 plasmid was constructed to verify the inhibitory effect of curdione on CRC.

**Results:**

A dose-dependent decrease in activity was observed in curdione-treated cells. Curdione increased the accumulation of reactive oxygen species in CRC cells and tumor tissues, greatly enhanced the levels of malondialdehyde, lipid hydroperoxide and Fe^2+^, and lowered the activity of glutathione. According to the qPCR and Western blot results, curdione promoted the expression of METTL14 and YTHDF2 in CRC cells and tissues, respectively, and decreased the expression of SLC7A11, SLC3A2, HOXA13, and glutathione peroxidase 4. Additionally, in animal experiments, the curdione-treated group showed severe necrosis of tumor cells, as displayed by HE staining. Furthermore, compared with the control group, levels of m6A modifying factors (namely, SLC7A11 and HOXA13) were increased in the tissues after drug intervention. METTL14 knockdown was followed by an increase in CRC cell activity and glutathione levels. However, the levels of reactive oxygen species, malondialdehyde, and iron ions decreased. The expression levels of SLC7A11, SLC3A2, HOXA13, and GPX4 were all increased after METTL14 knockdown.

**Conclusion:**

The results suggest that curdione induces ferroptosis in CRC by virtue of m6A methylation.

**Supplementary Information:**

The online version contains supplementary material available at 10.1186/s13020-023-00820-x.

## Introduction

With consideration of the data acquired, colorectal cancer (CRC) has the third and second highest incidence and fatality rates [1], respectively, severely threatening human life and health. Moreover, the incidence of CRC is higher in younger patients [2]. As displayed by growing evidence, ferroptosis is involved in CRC development [3, 4]. According to some studies [5–10], induction of cellular ferroptosis can inhibit CRC development. Hence, ferroptosis is deemed an important method for the inhibition of CRC development. The traditional Chinese medicine *Curcumae Rhizoma* is the dried rhizome of *Curcuma phaeocaulis* Val., *Curcuma kwangsiensis* S.G. Lee et. C. F. Liang or *Curcuma wenyujin* Y.H. Chen et C. Ling. Curdione, as the most abundant sesquiterpene in *Curcumae Rhizoma*, has activities including anti-platelet aggregation [11], contributing to the alleviation of adverse effects of chemotherapy and the promotion of apoptosis in liver cancer cells [12]. Nevertheless, little attention has been given to curdione as a means of combating CRC, and the underlying mechanism remains unknown. In this study, we aimed to further evaluate the mechanisms underlying curdione-induced ferroptosis in CRC, as well as its inhibitory effect on CRC.

In 2012, ferroptosis was first reported by Dixon et al. [13]. As a novel mode of cell death characterized by increased intracellular reduced iron and lipid peroxide accumulation, ferroptosis can induce cell death. In view of morphology, biochemistry, genetics, and function, ferroptosis differs from cell necrosis, apoptosis, and autophagy. Morphological features of ferroptosis include enhanced bilayer density, ruptured outer mitochondrial membranes, and mitochondrial crinkling. System XC- is an important pathway of ferroptosis. The cystine/glutamate reverse transporters (SLC7A11 and SLC3A2) are transporter proteins located in the cell membrane. The SLC7A11 subunit functions as an interchange between intracellular glutamate and extracellular cystine. By inhibiting SLC7A11 expression, ferroptosis can be induced. HOXA13 serves as a transcription factor, promoting SLC3A2 transcription [14]. High HOXA13 expression is associated with poor cancer survival, and it also plays a role in the promotion, growth, and therapy resistance of many malignancies [15, 16]. Inhibition of system Xc- decreases the cellular uptake of cystine, further leading to the reduced synthesis of glutathione (GSH), an important intracellular antioxidant factor. Glutathione peroxidase 4 (GPX4) is an inhibitor of lipid peroxidation, scavenging accumulated intracellular reactive oxygen species (ROS). Reduced GSH synthesis indirectly promotes ROS and lipid hydroperoxide (LPO) accumulation, resulting in ferroptosis. Ferroptosis is closely related to the methylation enzyme METTL14 [17]. Fan et al. have demonstrated that METTL14, by recognizing and binding SLC7A11 mRNA with YTHDF2, regulates the mRNA methylation of SLC7A11 and promotes its degradation [18]. Furthermore, METTL14 displays low expression in CRC and mediates m6A modification, thereby inhibiting CRC tumor metastasis [19].

m6A (N6-methyladenosine), a methylated form of the adenosine N6 site, was first discovered in the 1970s [20]. The presence of m6A modifications is crucial for RNA splicing, translation, and stability [21, 22]. During RNA methylation modification, there are three types of enzymes involved, namely, METTL3, METTL14, and WTAP (also known as methyltransferases and writers). Among them, METTL14 serves more as an oncogene regulating CRC development [19, 23]. Demethylases, called ‘‘erasers’’, contain FTO and ALKBH5. The m6A recognition proteins are referred to as ‘‘readers’’, including the YTH structural domain protein family and the HNRNP protein family [24], as well as the IGF2BP proteins [25]. According to the first study with radiolabeled tracers in 1978, YTHDF2 mediates the instability of m6A-containing mRNAs. Acting as one of the cytoplasmic m6A reading proteins, YTHDF2 recognizes bound m6A-methylated RNAs and recruits them to the cytoplasm for degradation [21]. The abnormal regulation of m6A modifications in RNAs identified in CRC tissues is greatly related to carcinogenesis, progression, and metastasis [26]. In addition, m6A regulators and m6A-associated RNAs might be novel biomarkers, prognostic correlates, and therapeutic targets.

Based on our previous experiments and literature studies, curdione increases the expression of METTL14 (a methylation transferase), enhances the methylation of XC-system subunit SLC7A11 mRNA and HOXA13 mRNA, lowers the stability of reading protein YTHDF2, and reduces HOXA13, causing a decline in another subunit of the XC-system SLC3A2 expression, thus activating ferroptosis.

## Materials and methods

### Materials

The CT26 murine-derived CRC cell line and SW480 human-derived colorectal cell line were both purchased from Cell Resource Centre, the Chinese Academy of Sciences. BALB/c nude male mice (7 weeks old) were obtained from Spelford (Beijing) Biotechnology Co., Ltd, with license number SCXK (Beijing) 2019–0010. Curdione was purchased from Macklin (C860637, Shanghai, China), with a purity exceeding 99%. Oxaliplatin was acquired from Sigma (O9512, Shanghai, China). Fetal bovine serum, dulbecco's modified eagle medium, trypsin digest and penicillin‒streptomycin combination were obtained from Gibco (America). METTL4 (abs100661, 1:1000) antibody was purchased from Absin. Antibodies against METTL3 (ab195352, 1:1000), YTHDF2 (ab275037, 1:1000) and GPX4 (ab125066, 1:1000) were purchased from Abcam. Antibodies against METTL14 (48,699, 1:1000), SLC7A11 (12,691, 1:1000), SLC3A2 (47,213, 1:1000), GAPDH (5174, 1:5000), and IgG (H + L) (rabbit, 14,708, 1:5000) were purchased from CST. The antibody against HOXA13 (CY8033, 1:1000) was acquired from Abways. SAB5600251 (SAB5600251, 1:500) was provided by Merck. A reactive oxygen species assay kit (S0033M) was provided by Beyotime. An Iron Assay Kit (MAK025) was obtained from Sigma. A malondialdehyde assay kit (BC0025), lipid hydroperoxide assay kit (BC5245), and reduced glutathione assay kit (BC1175) were purchased from Solarbio.

### Cell culture

Murine-derived CT26 cells and human-derived SW480 cells were routinely cultured (37 °C, 5% CO_2_) in DMEM complete medium containing 10% fetal bovine serum and 1% penicillin‒streptomycin in volume fractions. When the number of cells reached 70–80%, curdione, prepared from DMSO at different concentrations (12.5 μM, 25 μM, and 50 μM), was added to intervene CRC cells for 48 h.

### Plasmid transfection

The day before transfection, the cells were placed in growth medium (without antibiotics), thus ensuring 90–95% confluence at the time of transfection. With biosystems, the recombinant vector plasmid METTL14 shRNA was designed and constructed. To lower the expression of METTL14, the coding sequence of METTL14 was cloned into the vector pLV3ltr-ZsGreen-Puro-U6 by using BamH1 and EcoR1 as enzyme cleavage sites [27]. Considering the instructions of the Lip2000 transfection reagent (L7800, Solarbio), the interfering plasmid was transfected into CT26 cells. After transfection with 1 μg of the expression vector plasmid, the cells were cultured in an incubator for 48 h.

### Methyl thiazolyl tetrazolium (MTT) assay for cell viability

In the logarithmic development stage, cell suspensions were prepared by counting and processing CT26 cells. Successively, 96-well plates were obtained by counting and punching cell suspensions for culture. Various doses of curdione were used to treat the cells, thereby evaluating cell activity with MTT, DMSO reagent, and a microplate reader. (51119570, Thermo, Shanghai, China).

### Measurement of ROS by flow cytometry

After being exposed to low, medium, and high concentrations of curdione for 48 h, CT26 cells were stained with dichlorofluorescein diacetate (DCFH-DA, Beyotime) to assess the total quantity of intracellular ROS. Afterward, the cells were rinsed with phosphate-buffered saline (PBS) followed by treatment with 10 μM DCFH-DA for 30 min at 37 °C. Before flow cytometry, the cells were washed twice in PBS. The detailed steps were performed by referring to the manual offered by Beyotime Biotechnology (S0033M, Beyotime, Shanghai, China).

### Western blot

Total proteins were extracted from CRC cells or tissues, followed by separation with a 10% SDS‒PAGE system. Subsequently, the proteins were transferred to the membranes at a constant pressure of 100 V for 2 h (041BR121667, Bio-Rad) and blocked with 5% skimmed milk for 2 h at room temperature. After adding primary antibody, the membranes were incubated with primary antibodies overnight at 4 °C, washed three times with PBS, and incubated with secondary antibody corresponding to horseradish peroxidase (HRP). Afterward, the membranes were visualized with Gel imaging systems (Tanon-4600, Shanghai, China), and ImageJ software was employed to quantify the individual band intensity.

### ELISA

The recommended tissues and cells were homogenized (Scientz-48 L, Ningbo, China) in an ice bath by adding extracts in accordance with the manufacturer's procedure for the Fe^2+^ assay. The supernatant was centrifuged at 4 °C for 10 min, the absorbance value was measured on a microplate reader, and the amount of Fe^2+^ was calculated by applying the formula. In the same way as Fe^2+^, the contents of MDA, LPO and GSH were determined by complying with the kit instructions offered by the manufacturer of Solarbio.

### RNA extraction and quantitative real-time polymerase chain reaction (qRT‒PCR) assay

The TRIzol technique was adopted to extract total RNA from each group of CRC samples. Equipment (QuantStudio 5, ABI) was also used to identify the RNA concentration in all test samples, followed by reverse transcription and cDNA synthesis. Two microliters of cDNA was used to perform qPCR experiments. After 40 cycles, GAPDH was used as an internal reference gene, and the relative expression of the target gene was measured with the 2^−△△CT^ relative quantification approach. The primer sequences applied in the qPCR experiments are shown in Table 1.

**Table 1:** PCR primer sequences

Gene name	Primer sequences(5ʹ—3ʹ)
METTL4-F	TAGTTGCTGAGTGGCACTGG
METTL4-R	TCCAAATATTCCCCATCTGGCT
METTL14-F	GTAGCACAGACGGGGACTTC
METTL14-R	TTGGTCCAACTGTGAGCCAG
METTL3-F	ATCCCCAAGGCTTCAACCAG
METTL3-R	GGGTTGCACATTGTGTGGTC
GPX4-F	GGAGCCAGGGAGTAACGAAG
GPX4-R	GACGGTGTCCAAACTTGGTG
YTHDF2-F	CAGGCATCAGTAGGGCAACA
YTHDF2-R	GGACCGAAGCTTCTCCAACA
SLC7A11-F	TGGTCAGAAAGCCTGTTGTGT
SLC7A11-R	GCACGCCCTTAGGAGAGATG
SLC3A2-F	GGGCCTGGACTCTTCTCCTA
SLC3A2-R	CCTTCGTGAGGCTCCAGTTT
HOXA13-F	GAACGGCCAAATGTACTGCC
HOXA13-R	CGCCTCCGTTTGTCCTTAGT
PTGS2-F	AGTCCCTGAGCATCTACGGT
PTGS2-R GAPDH-F GAPDH-R	GCCTGCTTGTCTGGAACAAC GCCTCCTCCAATTCAACCCT CTCGTGGTTCACACCCATCA

### Animal treatments

The animal experiments were approved by the Ethics Committee of Nanjing University of Chinese Medicine, with ethics approval number A220607 for the use of animals in this study. A 200 μL CT26 cell suspension at a concentration of 0.5 × 10^7^/mL was injected into one side of the axilla of nude mice to generate a CT26 subcutaneous transplantation tumor model, followed by daily tumor development monitoring. Nude mice, successfully affected with CRC, were randomly divided into five groups, with six mice in each group and different doses of curdione for the administered groups. The five groups included one control group, one low-dose group (50 mg/kg curdione, dissolved in 10% DMSO), one medium-dose group (100 mg/kg curdione, dissolved in 10% DMSO), one high-dose group (200 mg/kg curdione, dissolved in 10% DMSO), and one positive group (oxaliplatin, 5 mg/kg) administered via tail vein injection. The tumor volume of the xenograft mice was measured every three days. After 22 days, the mice were sacrificed under anesthesia.

### Hematoxylin–eosin (HE) staining

The stripped CRC tumors were fixed with 4% paraformaldehyde solution, dehydrated (YD-12P, Shanghai, China), embedded (YD-6 L, Shanghai, China), and sectioned (RM2235), followed by routine HE staining (YD-700, Shanghai, China), microscopic observation (DM-1000, Hangzhou, China), and image acquisition for analysis (HS6, SDPTOP).

### Spot hybridization assay for detection of overall m6A expression levels

To isolate total RNA, TRIzol reagent was used. RNA was concentrated and diluted, followed by heating for five minutes at 95 °C to denature it. An nylon membrane (Amersham Hybond-N +) was loaded with 2 μL RNA. After UV cross-linking, the membrane was blocked with 5% skim milk for an hour at room temperature. Afterward, the membrane was treated with m6A antibody (1:500, Merck) overnight at 4 °C and then with secondary antibody for an hour. By applying a chemiluminescence technique, the signal was discovered following PBST washing (Bio-Rad). As a loading control, membranes were colored in 0.3 mol/L sodium acetate (pH 5.2) with 0.02% methylene blue.

### MeRIP-qPCR experiments

To explore m6A gene differentiation, the Magna MeRIPTM m6A kit was adopted for MeRIP, with the following procedures. RNA was extracted from CRC tissue and fragmented; a fraction of the RNA sample was employed as the input group, and the remainder of the RNA sample was regarded as the IP group. IP buffer and antibodies were added to the IP group, followed by incubation for 4 h in a vertical mixer at 4 °C. Afterward, the IP group was pretreated with magnetic beads and then incubated with produced Protein A/G magnetic beads in a vertical mixer at 4 °C for 1 h. After adsorption of the beads, the supernatant was removed. Subsequently, the wash buffer was added to the EP tube and gently blown into the tube. The tube was then washed for 5 min at 4 °C in a vertical mixer. The beads were adsorbed on a magnetic stand, followed by supernatant removal. After completing the previous process twice, 200 μL elution buffer was added to the input material, and then RNA extraction was performed by employing phenol‒chloroform. Finally, qPCR was adopted to detect identical amounts of reverse transcribed RNA.

### TUNEL staining

Apoptosis in SW480 cells was detected by employing the TUNEL kit. Following dewaxing at room temperature, soaking in gradient ethanol, rinsing with PBS, and incubation, adherent cells were cultured. Afterward, apoptosis was induced, anti-fluorescence quenching and sealing were performed, and the cells were blocked for microscopy and analysis.

### Statistical analysi*s*

GraphPad Prism 8.0 (GraphPad Software, Inc., San Diego, CA, USA) was applied to analyze and interpret the collected data, with the mean ± SD representing the results. One-way analysis of variance (ANOVA) was adopted to analyze the difference between the treatment group and the control group. All experiments were repeated at least three times. P < 0.05 was deemed statistically significant.

## Results

### Inhibition of CT26 cell activity by curdione

**Fig. 1:**
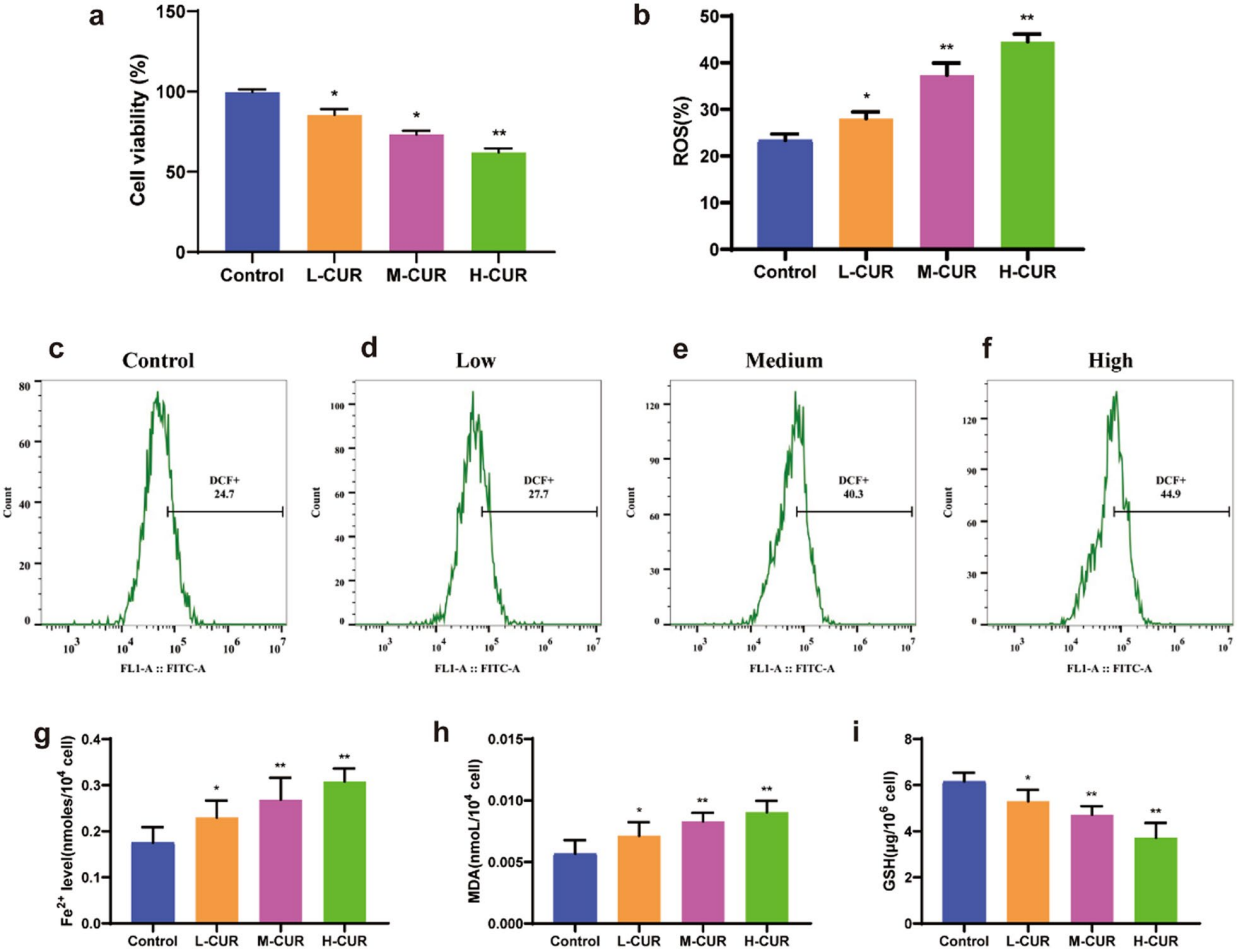
Curdione affects the activity of CT26 cells and causes changes in reactive oxygen species, Fe^2+^, MDA, and GSH. **a**. The cell viability of colorectal cancer CT26 cells was measured by MTT assay after 12.5 μM, 25 μM, and 50 μM curdione interventions for 48 h. **b**–**f**. The accumulation of reactive oxygen species was detected by flow cytometry after 12.5 μM, 25 μM, and 50 μM curdione interventions in CT26 cells for 48 h. **g**–**i**. Fe^2+^, MDA, and GSH levels in CT26 cells from different groups treated with curdione for 48 h were measured according to the manufacturer's instructions and ELISA kits. Values are expressed as the mean ± SD of three independent experiments. (n = 3) ^*^P < 0.05, ^**^P < 0.01, ^***^P < 0.001 vs. control group

After 48 h of treatment with different doses of curdione, the vitality of CT26 cells, a murine-derived CRC cell line, was assessed by MTT assay, thereby validating curdione's inhibitory efficacy on CRC cell line growth. Compared to control cells, CT26 viability decreased in a dose-dependent manner, and the lowest cell survival was reported at 50 μM (*P* < 0.01) (Fig. 1(a)). curdione drastically decreased the viability of CRC cells in vitro.

### Increase in the accumulation of reactive oxygen species in CRC cells due to curdione

As ferroptosis was accompanied by an increase in the number of ROS and an expansion of lipid peroxides, the DCFH-DA fluorescent probe was employed to determine the level of intracellular ROS. Compared to the control group (Fig. 1(b–f)), the low-dose (*P* < 0.5), medium-dose (*P* < 0.01), and high-dose (*P* < 0.001) groups exhibited considerably increased fluorescence intensity of ROS. This indicates that curdione can tremendously promote intracellular ROS production.

### *Influence of curdione on Fe*^*2*+^*, MDA, LPO, and GSH activities in CRC cells*

With the reagent kit, GSH, MDA, LPO, and divalent iron ions in the cells were measured, with results presented in Fig. 1(g–i). Compared with the control group, curdione at 12.5 μM (*P* < 0.5), 25 μM (*P* < 0.01), and 50 μM (*P* < 0.01) dramatically lowered the concentration of GSH while increasing the levels of MDA and divalent iron ions in a dose-dependent manner. Curdione enhanced ferroptosis in cells by promoting lipid peroxidation, increasing the accumulation of the lipid peroxidation product MDA, and decreasing the intracellular GSH concentration, which are all indications of ferroptosis. Furthermore, with an increase in the concentration of curdione, the LPO level was promoted in CT26 cells (Additional file 1: Figure S1f). Additionally, the same results were demonstrated in SW480 cells (Additional file 1: Figure S1a–d), and curdione induced ferroptosis in CRC cells. According to the results, there were remarkable differences in all groups (*P* < 0.01).

### Impact of curdione on the expression of the proteins METTL14, YTHDF2, SLC7A11, SLC3A2, HOXA13, and GPX4 in CRC cells

**Fig. 2:**
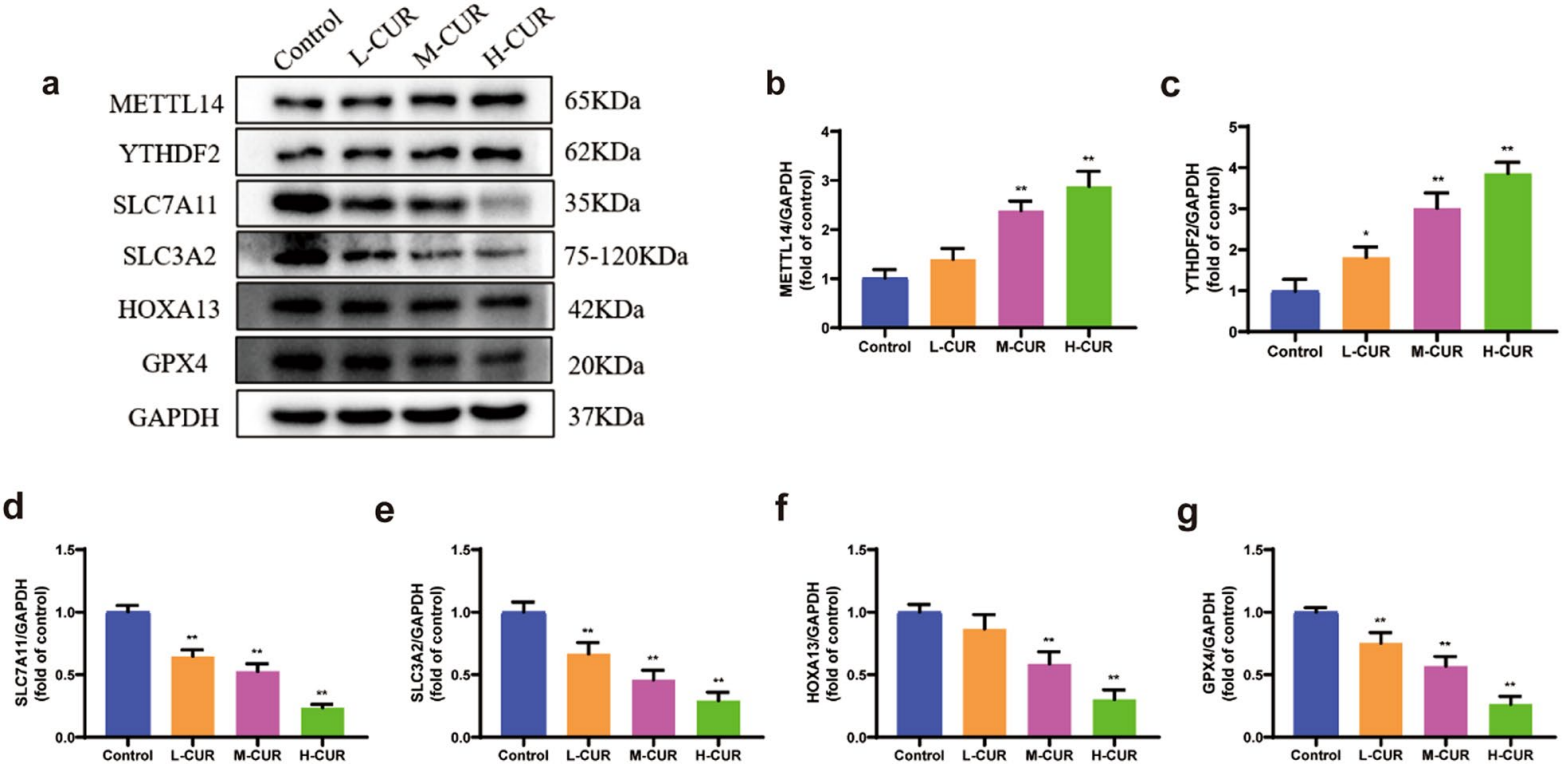
Curdione promoted the expression of METTL14 and YTHDF2 and reduced the expression of SLC7A11, SLC3A2, HOXA13, and GPX4. **a**–**g**. The protein levels of METTL14, YTHDF2, SLC7A11, SLC3A2, HOXA13, and GPX4 in CT26 cells were measured by Western blotting after 12.5 μM, 25 μM, and 50 μM curdione interventions for 48 h. Values are expressed as the mean ± SD of three independent experiments. (n = 3) ^*^P < 0.05, ^**^P < 0.01, ^***^P < 0.001 vs. control group

As depicted in Fig. 2(a–g), the relative expression of METTL14 and YTHDF2 was promoted in the curdione group compared to the control group, and the protein expression exhibited substantial increases at 25 μM and 50 μM (*P* < 0.01). The relative expression levels of SLC7A11, SLC3A2, HOXA13, and GPX4 were reduced in a dose-dependent manner (*P* < 0.01). Thus, 50 μM curdione was selected for further mechanistic study.

### Effect of curdione on CRC xenograft mice

**Fig. 3:**
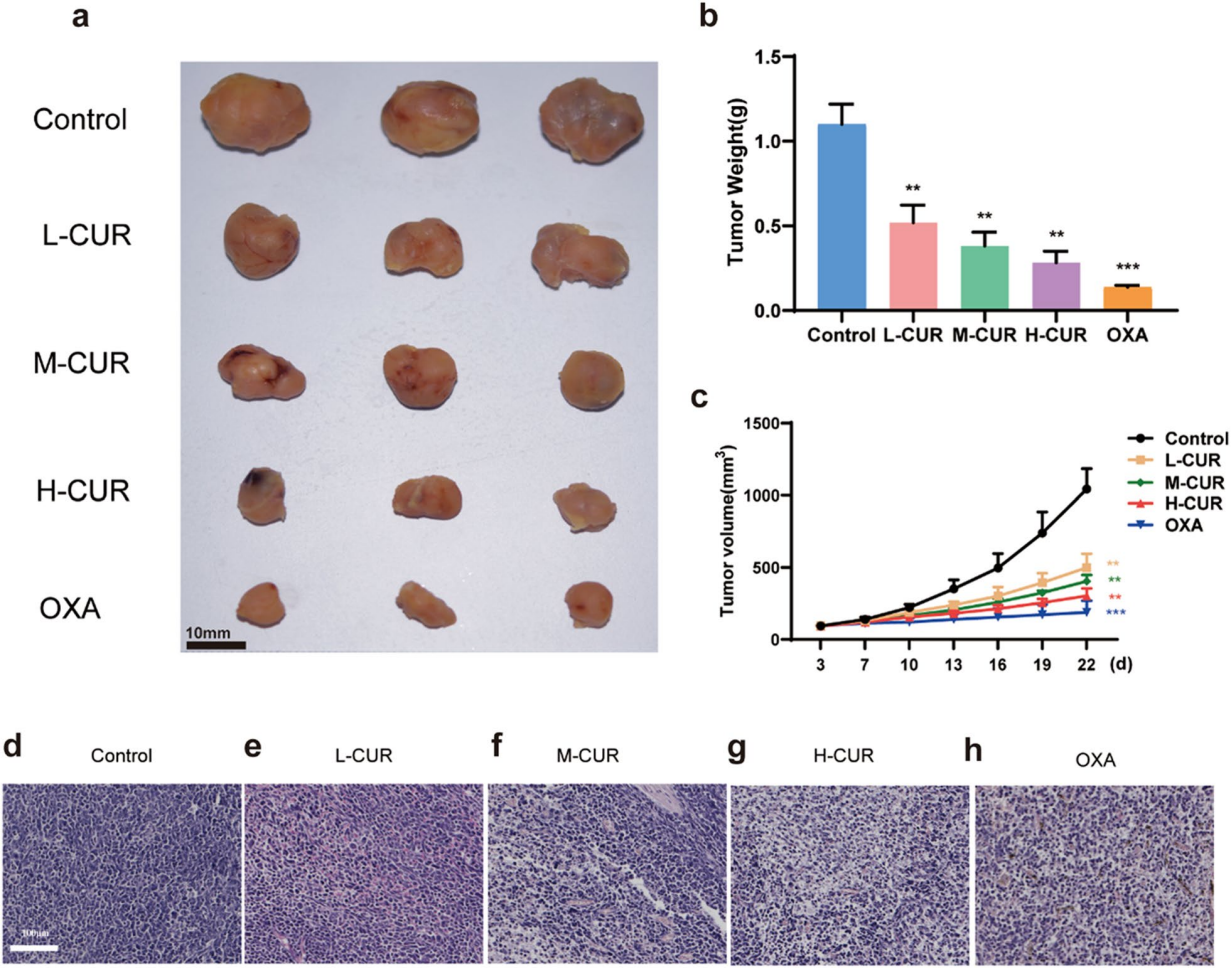
Curdione inhibited tumor formation in colorectal cancer xenograft mice. **a**–**c**. After 22 days of drug administration (50 mg/kg, 100 mg/kg, 200 mg/kg curdione, dissolved in 10% DMSO and 5 mg/kg oxaliplatin), xenograft mice were dissected, and tumor mass and volume were monitored. (6 xenograft mice in each group) **d**–**h**. Tumor tissues of xenograft mice in the model group, curdione low-dose, medium-dose, and high-dose groups, and oxaliplatin-positive group were subjected to hematoxylin–eosin staining. Morphological changes in tumor cell arrangement density, tumor cytoplasm, and nuclei were observed. Values are expressed as the mean ± SD of three independent experiments. (n = 3) ^*^P < 0.05, ^**^P < 0.01, ^***^P < 0.001 vs. control group

To verify the effect of curdione on CRC mice, different doses of curdione were administered into the tail vein after successful modeling. Following the harvest of tumors, the mass and volume were measured after 22 days. According to the in vivo tumorigenesis assay, the volume and mass tumor suppression rates were higher in all administered groups than in the control group (*P* < 0.01) (Fig. 3a–c). According to HE staining observations, rather than showing cells with severe necrosis, the model group exhibited tightly arranged and dense cells, with full cytoplasm, enlarged nuclei, and obvious cellular anisotropy. In the high-dose curdione group and oxaliplatin group (*P* < 0.001), the cells displayed more obvious wrinkling and nuclear chromatin border set, accompanied by necrosis and fibrous hyperplasia, as well as reduced cellular arrangement density. Considering that the therapeutic effect of the high dose of curdione was close to that of oxaliplatin with no side effects, the oxaliplatin group was not subsequently added (Fig. 3d–h). Curdione acts on ferroptosis, but the positive drug does not act on ferroptosis, so there is little point in studying the effect of the positive drug on ferroptosis, and the results would differ significantly from those of curdione, so the positive drug group was removed from the later experiments. (Fig. 3d–h).

### *Comparison of Fe*^*2*+^*and MDA levels and GSH activity in various groups of CRC mice*

**Fig. 4:**
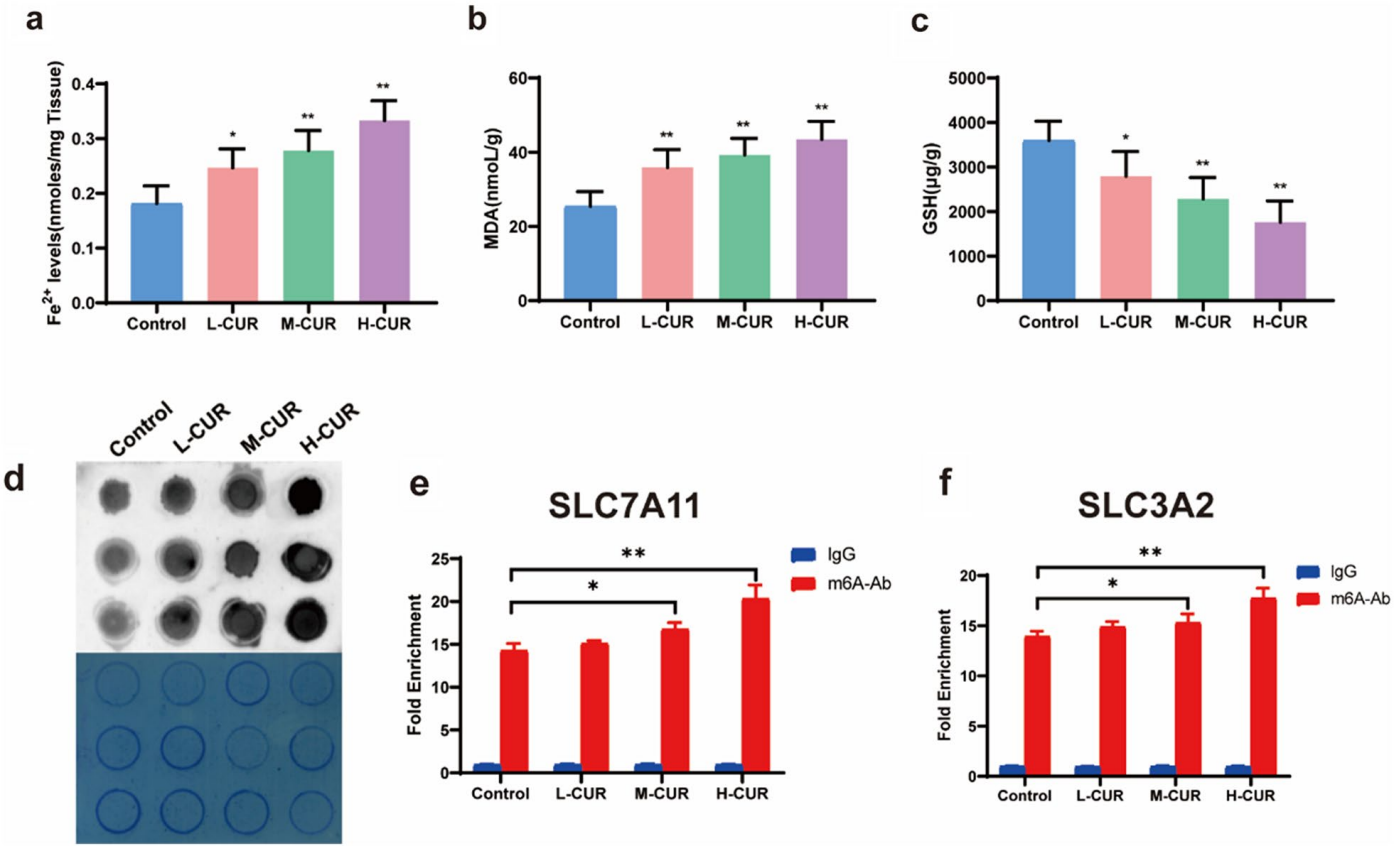
Curdione caused changes in ferroptosis and increased m6A modification in CRC xenograft mouse tissues, promoting m6A levels in SLC7A11 and HOXA13. **a**–**c**. ELISA kits for the determination of Fe^2+^, MDA, and GSH in tumor tissues of xenograft mice according to the manufacturer’s protocol. **d**. Spot blotting was used to determine the overall m6A levels of RNA in the four groups. The darker the color of the spot, the higher the m6A level. **e**–**f**. The effect of curdione on the m6A modification levels of SLC7A11 and HOXA13 was investigated using MeRIP-qPCR. Values are expressed as the mean ± SD of three independent experiments. (n = 3) ^*^P < 0.05, ^**^P < 0.01, ^***^P < 0.001 vs. control group

The surplus of iron ions is one of the important mechanisms of ferroptosis. As observed, the levels of iron ions and peroxides exhibited changes in the CRC tissues of the four groups of mice. Considerably increased levels of iron ions, MDA and LPO and remarkably lower GSH were revealed in the curdione low-dose (*P* < 0.05, *P* < 0.01), medium-dose (*P* < 0.01), and high-dose (*P* < 0.01) groups compared to the control group (Fig. 4a–c).

### Increase in m6A modification in CRC and promotion of m6A levels in SLC7A11 and HOXA13 due to curdione

Spot blotting was performed on CRC tissues and SW480 cells, thereby determining the overall m6A levels of RNA in the four groups, with the results exhibited in Figs. 4d, Additional file 1: Figures S1e. Curdione intervention enriched higher levels of m6A, and it was observed that the higher dose group had darker spots, indicating that more m6A-modified RNA was bound. To explore m6A gene differentiation, MeRIP was conducted by employing the Magna MeRIPTM m6A kit. With MeRIP-qPCR experiments, the effect of curdione on the m6A modification levels of SLC7A11 and HOXA13 was investigated (Fig. 4e–f). When SLC7A11 and HOXA13 were recruited with m6A-specific antibodies, their mRNA abundance exhibited a remarkable increase. Significance was stronger in the high-dose curdione group (*P* < 0.01) than in the medium-dose curdione group (*P* < 0.05).

### Effect of curdione on the protein expression of METTL3, METTL4, METTL14, SLC3A2, SLC7A11, HOXA13, YTHDF2 and GPX4 in CRC mouse tissues

**Fig. 5:**
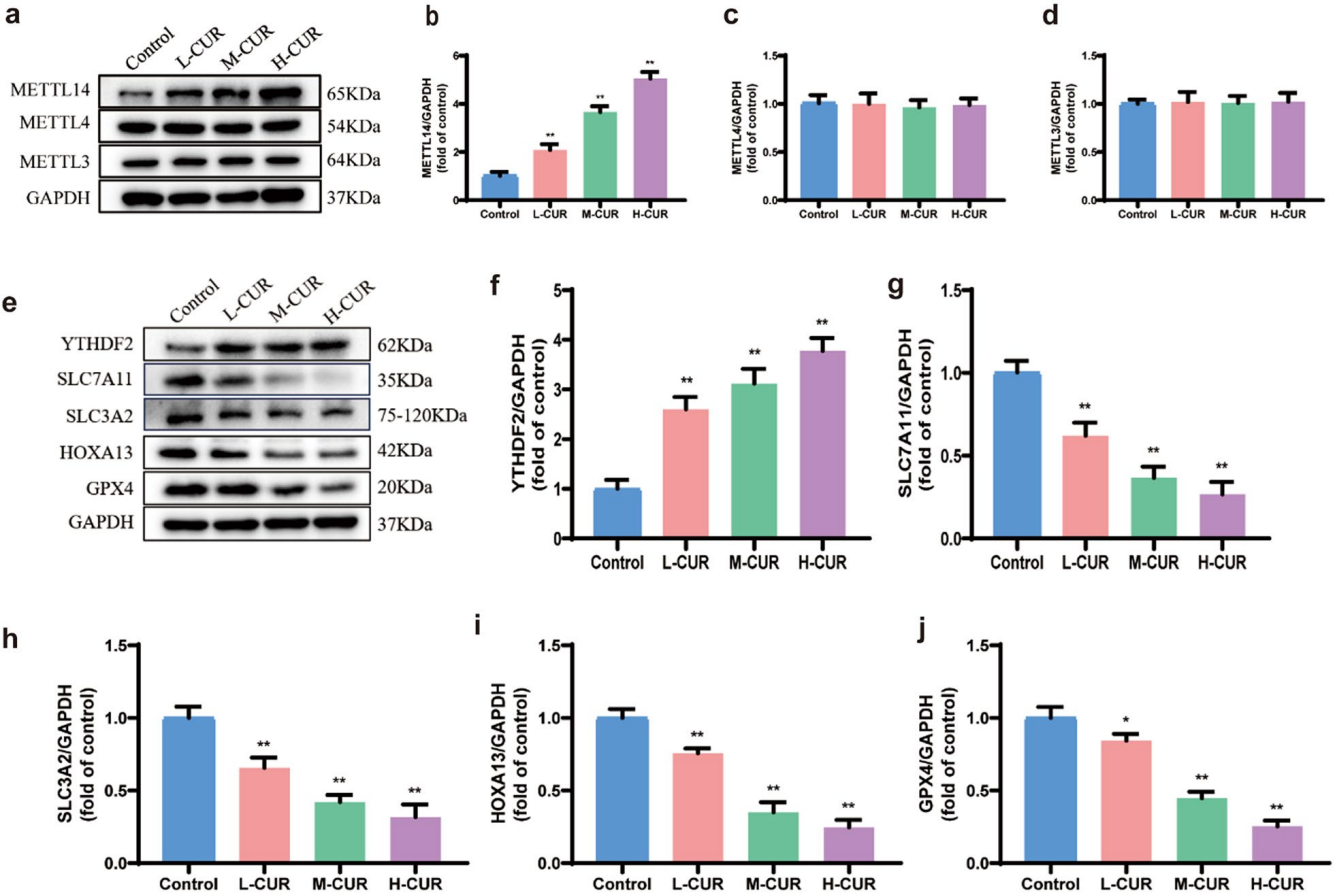
Curdione promoted the expression of METTL14 and YTHDF2 and reduced the expression of SLC7A11, SLC3A2, HOXA13, and GPX4. Mice were administered with L-CUR (50 mg/kg), M-CUR (100 mg/kg), and H-CUR (200 mg/kg) for 22 days. **a**–**j**. The protein levels of METTL3, METTL4, METTL14, YTHDF2, SLC7A11, SLC3A2, HOXA13, and GPX4 in CRC xenograft mice were measured by Western blotting. Values are expressed as the mean ± SD of three independent experiments. (n = 3) ^*^P < 0.05, ^**^P < 0.01, ^***^P < 0.001 vs. control group

According to Fig. 5, the expression levels of the methylation transferases METTL3 and METTL4 in the various dose groups exhibited no remarkable difference from those in the control group, regardless of the dose. The methylation transferase METTL14 exhibited great changes, with its expression being promoted with increasing doses. Curdione at low, medium, and high doses obviously increased METTL14 expression (*P* < 0.01), as displayed by the bar graph, with CRC mice administered a high dose of 50 mg/kg intervention having nearly five times more METTL14 expression compared to the control group. Curdione treatment increased the expression of YTHDF2, a reading protein related to m6A, particularly (*P* < 0.01) in a dose-dependent manner. As SLC7A11 and SLC3A2 are important components of the antioxidant system cysteine glutamate transporter receptor (system XC-), the reduction in HOXA13 decreased the expression of another subunit of the XC- system, SLC3A2, thereby activating ferroptosis. According to the graph, the protein expression levels of SLC7A11, SLC3A2, and HOXA13 were measured in comparison to the control. The expression of all three proteins showed a remarkable decrease with increasing dose when compared to the control (*P* < 0.01). Additionally, the expression of GPX4 was measured and was obviously reduced by curdione intervention (*P* < 0.01).

### Effect of curdione on the mRNA expression of METTL3, METTL4, METTL14, SLC3A2, SLC7A11, HOXA13, YTHDF2, GPX4 and PTGS2 in CRC mouse tissues

**Fig. 6:**
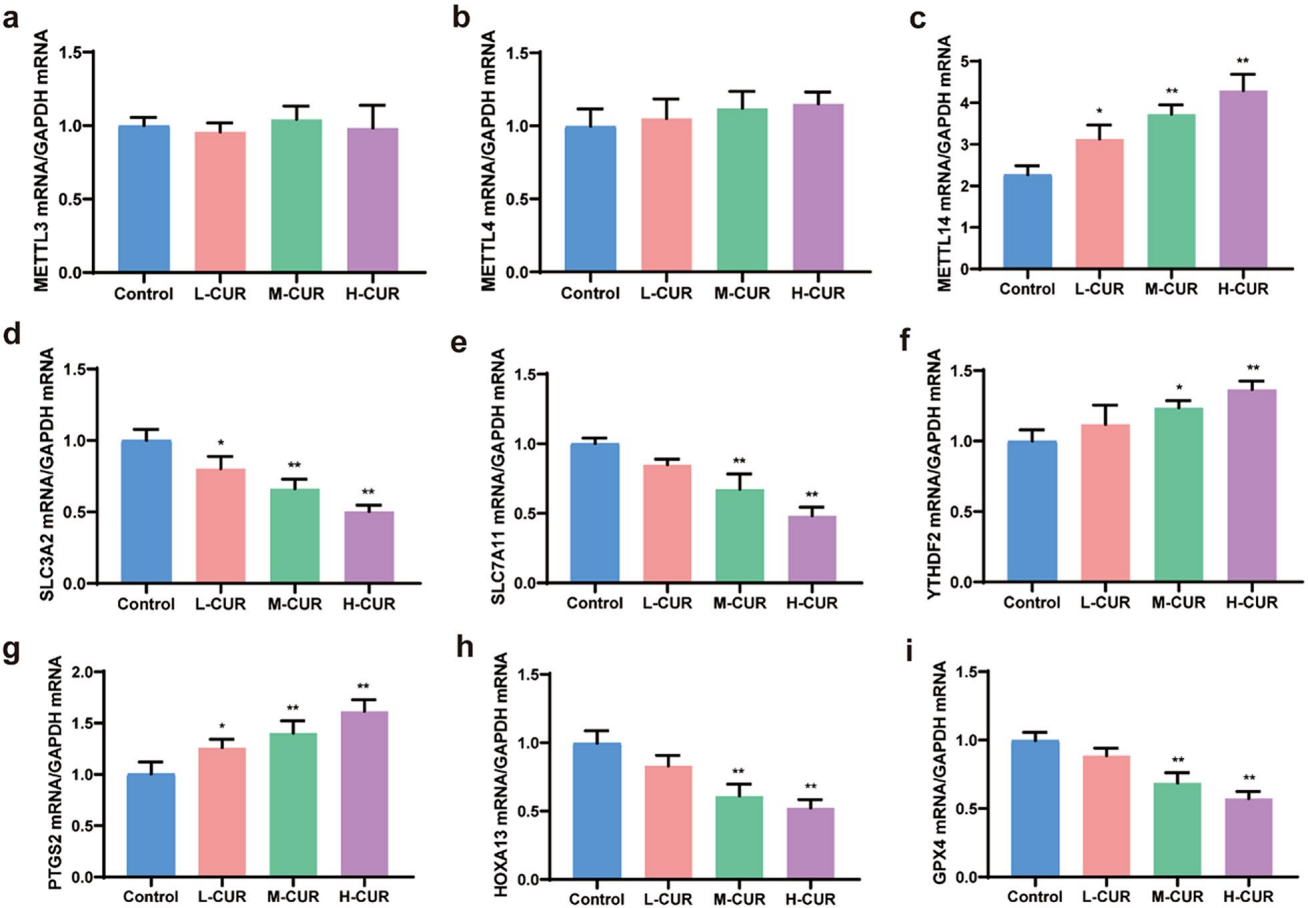
Curdione promoted the expression of METTL14, YTHDF2, and PTGS2 and reduced the expression of SLC7A11, SLC3A2, HOXA13, and GPX4. Mice were administered with L-CUR (50 mg/kg), M-CUR (100 mg/kg), and H-CUR (200 mg/kg) for 22 days. **a**–**i**. The mRNA levels of METTL3, METTL4, METTL14, SLC3A2, SLC7A11, HOXA13, YTHDF2, GPX4, and PTGS2 in CRC tissues were measured by qRT-PCR. Values are expressed as the mean ± SD of three independent experiments. (n = 3) ^*^P < 0.05, ^**^P < 0.01, ^***^P < 0.001 vs. control group

To determine whether ferroptosis played a role in the therapeutic impact of curdione in CRC mice, qPCR was adopted to assess the mRNA levels of METTL3, METTL4, METTL14, SLC3A2, SLC7A11, HOXA13, YTHDF2, GPX4, and PTGS2 in the tissues of four groups of CRC mice, as shown in Fig. 6. According to the results, the expression levels of METTL3 and METTL4 displayed no change in the administered group compared to the control group, which was similar to the results of Western blot. The expression of the methylation reading protein YTHDF2 was markedly increased with medium and high curdione dosages (*P* < 0.05, *P* < 0.01). SLC7A11, SLC3A2, and HOXA13 mRNA levels exhibited considerable increases (*P* < 0.05, *P* < 0.01). Compared to the control group, GPX4 mRNA expression levels were considerably lower (*P* < 0.01). As PTGS2 is also involved in ferroptosis pathology [28, 29], PTGS2 expression and activity were greatly enhanced after ferroptosis in cells stimulated by RSL3 or erastin. As a result, it was included in the qRT‒PCR experiment, and the increased mRNA expression level of PTGS2 was discovered with increasing medication concentration (*P* < 0.05, *P* < 0.01).

### Increase in m6A modification of the XC system and induction of ferroptosis by upregulating METTL14 expression due to curdione

**Fig. 7:**
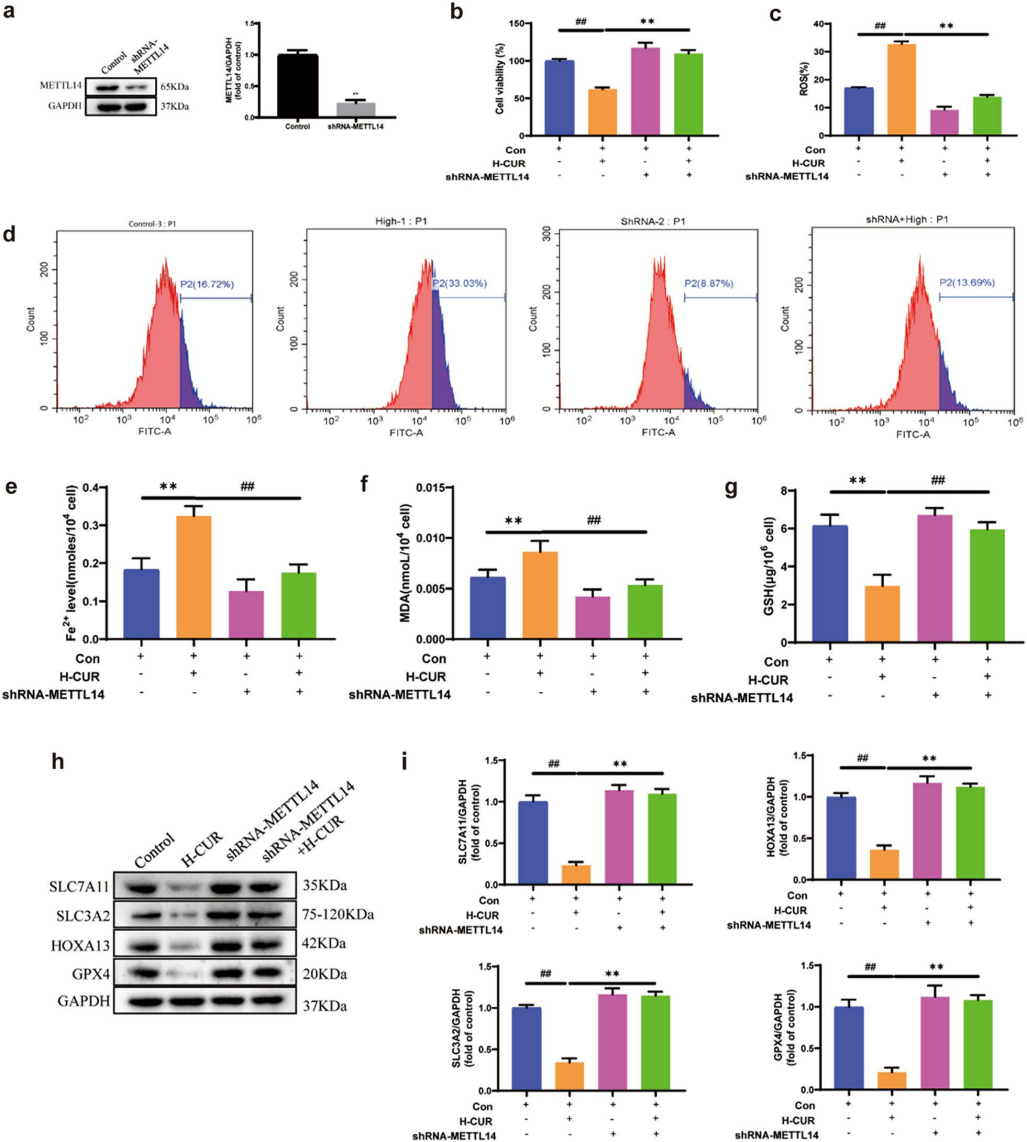
Curdione promoted ferroptosis in colorectal cancer through the upregulation of METTL14. **a**. The knockdown efficiency of METTL14 after 48 h of transfection of METTL14 shRNA CT26 cells was confirmed by Western blotting. ^**^P < 0.01 vs. control group. **b**. Different concentrations (12.5 μM, 25 μM, and 50 μM) of curdione prepared from DMSO were added to CRC cells for 48 h. MTT assay for each group of transfected cell activities. ^##^*P* < 0.01 H-CUR group vs. control group. ^**^P < 0.01 H-CUR + shRNA-METTL14 group vs. H-CUR group. **c**–**d**. Flow cytometry for each group of transfected cell ROS. Curdione decreased the production of intracellular ROS. ^##^*P* < 0.01 H-CUR group vs. control group. ^**^P < 0.01 H-CUR + shRNA-METTL14 group vs. H-CUR group. **e**–**g**. ELISA kits for the determination of Fe^2+^, MDA, and GSH in transfected cells. ^**^P < 0.01 H-CUR group vs. control group. ^##^*P* < 0.01 H-CUR + shRNA-METTL14 group vs. H-CUR group. **h**–**i**. The protein levels of SLC7A11, SLC3A2, HOXA13, and GPX4 in METTL14 knockdown CRC cells were detected by Western blotting. GAPDH was used as a control. Values are expressed as the mean ± SD of three independent experiments. (n = 3) ^##^*P* < 0.01 H-CUR group vs. control group. ^**^P < 0.01 H-CUR + shRNA-METTL14 group vs. H-CUR group

The shRNA-METTL14 was transfected into CT26 cells, with the transfection effect shown in Fig. 7(a). Control, high-dose, shRNA-METTL14, and shRNA-METTL14 + high-dose groups were formed. Cell activity was remarkably lower in the high-dose group than in the control group (*P* < 0.01) and notably higher in the shRNA-METTL14 + high-dose group than in the high-dose group. (*P* < 0.01), as shown in Fig. 7(b). Thus, the knockdown of METTL14 expression increased CT26 cell activity, according to previous studies reporting the promotion of CRC development by knockdown of METTL14 [6]. In the ROS test, the cellular ROS content was substantially greater in the high-dose group than in the control group (*P* < 0.01). The same high-dose curdione therapy was administered, and knockdown of METTL14 led to a notable decrease in ROS content (*P* < 0.01). As displayed in Fig. 7c–d, the shRNA-METTL14 + high-dose group notably decreased the cellular ROS concentration compared to the high-dose group (*P* < 0.01). With ELISA kits, the cellular Fe^2+^ and MDA contents of each group were measured, as well as the GSH activity. Compared to the control group, the addition of curdione at a concentration of 50 μM increased Fe^2+^ and MDA contents while decreasing GSH activity (*P* < 0.01). This conformed to earlier findings. Nonetheless, after knocking down METTL14, the levels of Fe^2+^ and MDA in the cells remained considerably decreased as predicted, whereas GSH activity was notably elevated in the shRNA-METTL14 + high-dose group compared to the high-dose group (*P* < 0.01). From another perspective, by knocking down the methylation transferase METTL14, Fe^2+^ and MDA levels were reduced, and GSH activity was increased, as displayed in Fig. 7e–g. According to the Western blot findings, knockdown of METTL14 boosted the expression of SLC7A11, HOXA13, SLC3A2, and GPX4. Compared to the high-dose group, SLC7A11, HOXA13, SLC3A2, and GPX4 expression was remarkably higher in the shRNA-METTL14 + high-dose group (*P* < 0.01), as shown in Fig. 7h–i. The same results were presented in SW480 cells with knockdown of METTL14. For details, please see Additional file 1: Fig S3 in the Supplementary Material. As shown by the results presented for both CRC cell lines, curdione induces CRC ferroptosis via m6A modification of the XC system and the methylation transferase METTL14.

### Induction of apoptosis by curdione, with its apoptosis inhibitor having no effect on ferroptosis

As reported, curdione can induce the onset of apoptosis [12, 30]. Another TUNEL staining experiment was conducted with SW480 cells to detect apoptosis. According to the fluorogram, apoptosis was evident in the cells upon intervention with high doses of curdione (*P* < 0.01), as shown in Additional file 1: Figure S2a, b. Afterward, further investigation was conducted to determine whether the level of curdione-induced ferroptosis was altered by treatment with the apoptosis inhibitor Z-VAD-FMK. As displayed in Additional file 1: Figure S2(c–f), cells were treated separately with 50 μM curdione and 10 μM Z-VAD-FMK or in combination, and the levels of Fe^2+^, MDA, LPO and GSH were measured. Our results indicate that curdione promoted the development of ferroptosis (*P* < 0.01). However, when combined with Z-VAD-FMK, there was no statistically significant difference. Nonetheless, a significant difference was observed between the Z-VAD-FMK group and the curdione + Z-VAD-FMK group (*P* < 0.01). Notably, the combined treatment group exhibited higher levels of ferroptosis, suggesting that apoptosis does not affect curdione-induced ferroptosis.

## Discussion

CRC is a highly fatal malignancy responsible for causing approximately nine million deaths annually, as reported by the Lancet [31]. In recent years, the utilization of Chinese medicine for the treatment of CRC has garnered the attention and recognition of medical experts and scholars worldwide. *Curcumae Rhizoma*, a Chinese medicine, has anticancer effects against a variety of cancers. The remarkable effects of its various active ingredients in inhibiting CRC development have been confirmed by many studies [32–35]. Nonetheless, there are few studies on the effect of curdione, an active ingredient, on CRC. Studies have mainly concentrated on breast cancer, kidney cancer, and pulmonary fibrosis [36]. There is also a gap in the research on CRC ferroptosis. In this study, curdione not only greatly decreased the development of CRC tumors and cells in vivo and in vitro but also triggered ferroptosis in CRC.

**Fig. 8:**
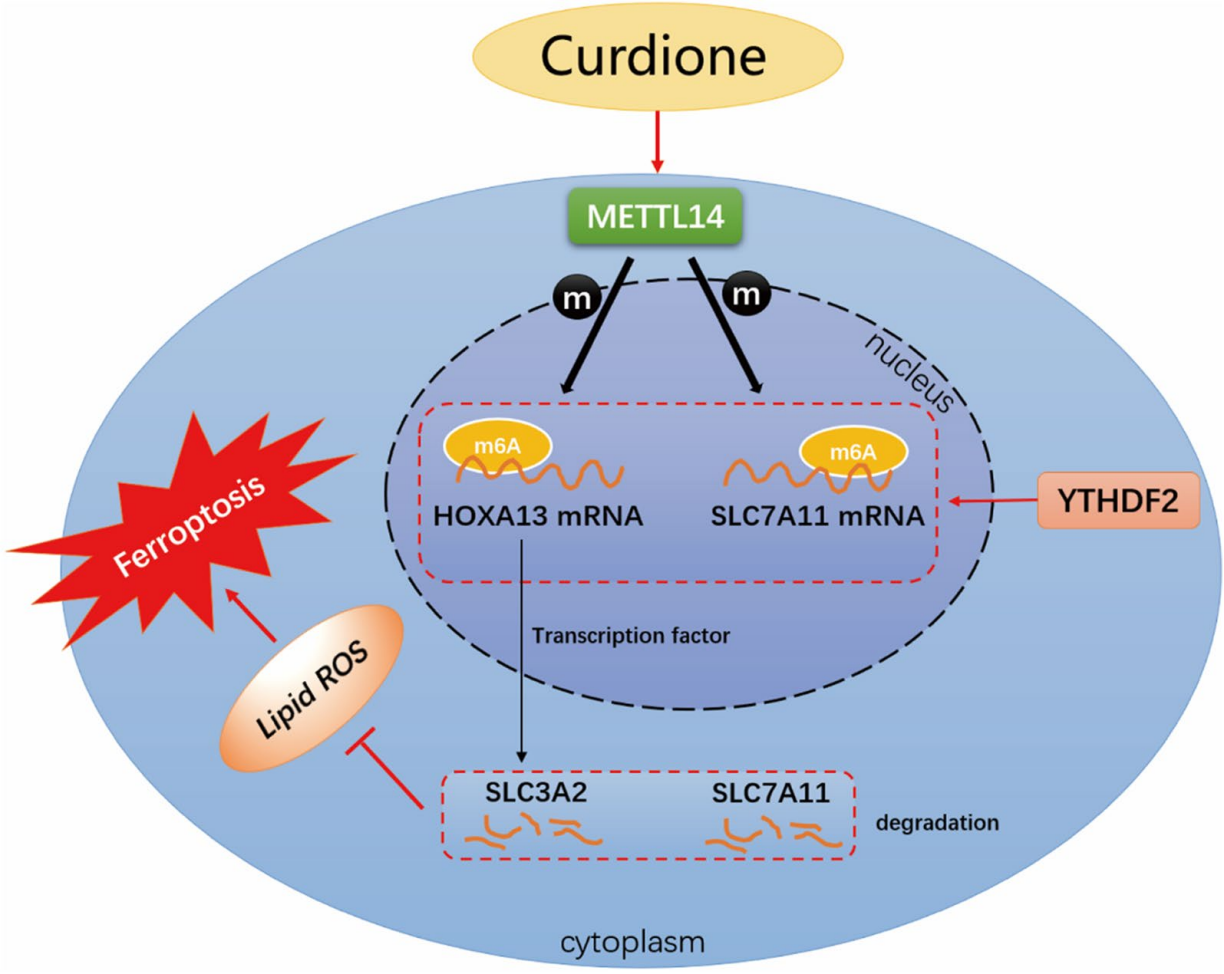
Curdione regulated ferroptosis in colorectal cancer via N6-methyladenosine. Curdione upregulated the methylation transferase METTL14, which increased m6A modification in SLC7A11 and SLC3A2 mRNAs. The m6A reader YTHDF2 then promoted the degradation of SLC7A11 and SLC3A2 mRNAs in a m6A-dependent manner, reducing their stability, which triggered reactive oxygen species accumulation and ultimately led to CRC ferroptosis

As a kind of necrosis induced by extramitochondrial lipid peroxidation, ferroptosis is primarily driven by iron-dependent ROS growth [37]. Ferroptosis may be an adaptive process that has considerable importance for the eradication of carcinogenic cells [38]. As suggested by a growing number of recent studies, clinical treatment of CRC may be facilitated by increasing intracellular Fe^2+^ and ROS, decreasing GSH levels in CRC cells or inactivating GPX4. However, inhibition of ferroptosis may result in tumor progression and treatment resistance in CRC [39]. A number of drugs targeting molecules in the ferroptosis pathway have been demonstrated to be effective in CRC treatment [7, 40]. Hence, it is a popular strategy to use drugs to induce ferroptosis in cancer. Chinese medicine is an important source for the discovery of anticancer drugs and has great potential to be exploited. As indicated by available reports, artemisinin, Astragalus membranaceus, curcumin and Dendrobium are capable of inducing ferroptosis [41]. Nevertheless, as revealed by our study for the first time, curdione induced ferroptosis in CRC by increasing the expression of METTL14 and YTHDF2. First, curdione greatly decreased the activity of CRC cells and decreased the tumor weight of CRC xenograft mice. Second, ferroptosis-related factors were examined in vitro and in vivo. According to the results, curdione not only enhanced the development of ferroptosis in addition to CRC but also elevated the expression of METTL14 and YTHDF2, with minimal impact on METTL3 and METTL4. METTL14 knockdown was demonstrated to enhance carcinogenesis and metastasis in vivo while increasing CRC cell proliferation and invasive potential in vitro [42]. Low expression of METTL14 promotes CRC metastasis in vitro and in vivo [23]. According to our study, curdione could increase METTL14 expression, so the final step of validation was performed based on the results of the previous cell experiments, namely, the construction of shRNA-METTL14 plasmids transfected into CRC cells, thereby investigating whether curdione promotes ferroptosis in CRC via METTL14. As demonstrated by the results, METTL14 knockdown boosted the survival rate of CRC cells while decreasing ROS, confirming the idea that curdione controlled ferroptosis in CRC via m6A methylation. The specific mechanism diagram is displayed in Fig. 8. Our experimental results strengthen the critical position and research value of METTL14 in CRC.

This study discovered a novel component (curdione) for CRC treatment in vitro and in vivo, elucidating that curdione induces ferroptosis by upregulating the expression of METTL14 and YTHDF2 via N6-methyladenosine. These results provide a reference or a new idea for the study of ferroptosis in CRC or other cancers.

However, it is important to acknowledge the limitations of this study. It has been proposed that ROS drive apoptosis and ferroptosis [43]. Ferroptosis plays a role in apoptosis triggering cell death [44]. Curdione, by combining with docetaxel, triggers ROS production and enhances docetaxel-induced apoptosis [45]. Therefore, it is speculated that there may be a link between curdione-induced ferroptosis and apoptosis, whether they act synergistically or do not interfere with each other. As found in some of our experiments, apoptosis had no influence on the induction of ferroptosis due to curdione. However, this result only represents a preliminary study, and its specific molecular mechanisms need to be meticulously designed, thereby further investigating whether apoptosis is affected by ferroptosis in the presence of pharmacological intervention. Furthermore, further in-depth study is needed on the anticancer mechanism of curdione.

## Conclusion

In conclusion, our study provides experimental evidence that curdione regulates ferroptosis in colorectal cancer by m6A methylation, revealing that curdione might be a promising treatment for colorectal cancer.

### Supplementary Information

**Additional file 1: Fig. S1.** Curdione induces ferroptosis in colorectal cancer. **Fig. S2.** Curdione-induced apoptosis does not affect the induction of ferroptosis. **Fig. S3.** Curdione promoted ferroptosis in colorectal cancer through the upregulation of METTL14.

## Data Availability

Data will be made available on request.

## Abbreviations

GPX4: Glutathione peroxidase 4
GSH: Glutathione
MDA: Malondialdehyde
H-CUR: High dosage curdione
HOXA13: Homeobox A13
L-CUR: Low dosage curdione
LPO: Lipid hydroperoxide
M-CUR: Middle dosage curdione
METTL14: Methyltransferase 14
METTL3: Methyltransferase 3
METTL4: Methyltransferase 4
OXA: Oxaliplatin
PTGS2: Prostaglandin-endoperoxide synthase 2
ROS: Reactive oxygen species;
SLC3A2: Solute carrier family 3 member 2
SLC7A11: Solute carrier family 7 member 11
YTHDF2: YTH N6-methyladenosine RNA binding protein F2
