# Supplementary material for: Curdione induces ferroptosis mediated by m6A methylation via METTL14 and YTHDF2 in colorectal cancer
Source: Chin Med. 2023 Sep 21;18:122. doi: 10.1186/s13020-023-00820-x (PMC10512537; doi:10.1186/s13020-023-00820-x)
Supplement: Supplementary file 1 — Additional file 1: Fig. S1. Curdione induces ferroptosis in colorectal cancer. Fig. S2. Curdione-induced apoptosis does not affect the induction of ferroptosis. Fig. S3. Curdione promoted ferroptosis in colorectal cancer through the upregulation of METTL14. [file 13020_2023_820_MOESM1_ESM.docx]

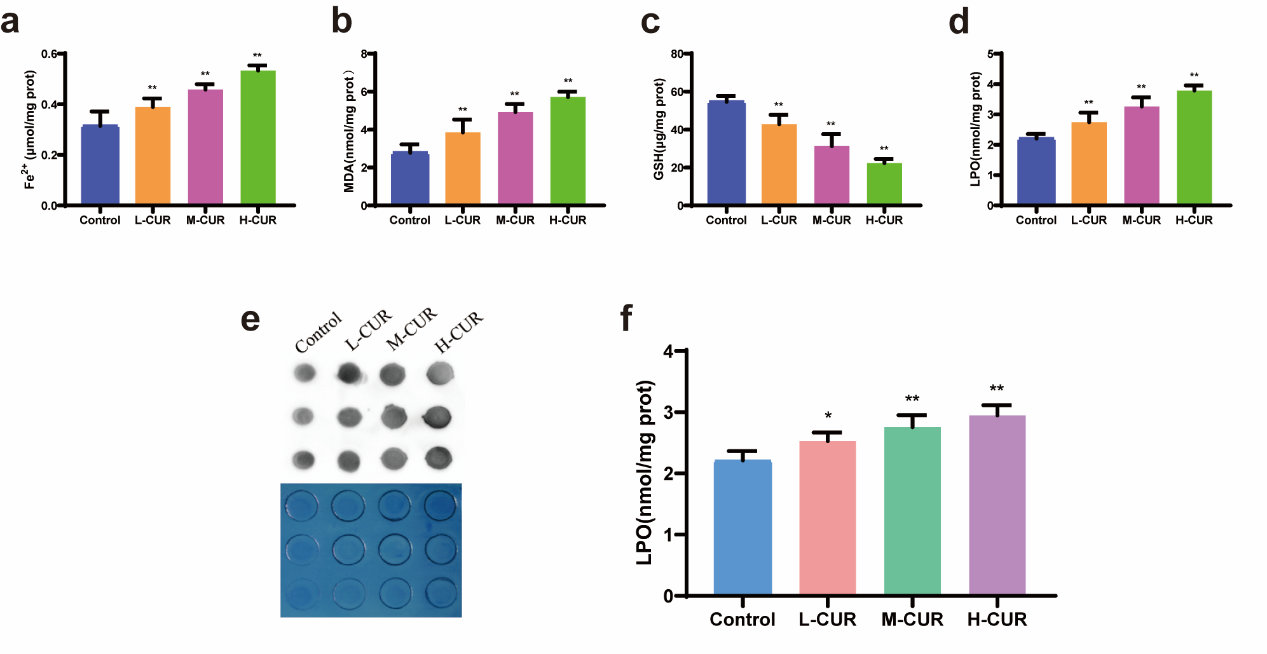


**Fig. S1**

**Fig. S1 Curdione induces ferroptosis in colorectal cancer.** SW480 cells were treated with 12.5 μM, 25 μM, and 50 μM curdione for 48 h. a-d. The levels of Fe^2+^, MDA, GSH, and LPO in different groups of curdione-treated SW480 cells were assayed according to the manufacturer's instructions and ELISA kits. Values are expressed as the mean ± SD of three independent experiments. (n=3) ^**^*P* < 0.01 vs. control group. e. Spot blotting was used to determine the overall m6A levels of RNA in four groups of SW480 cells. The darker the color of the spot, the higher the m6A level. f. LPO levels in each group were detected. Values are expressed as the mean ± SD of three independent experiments. (n=3)^*^*P* < 0.05, ^**^*P* < 0.01 vs. control group.


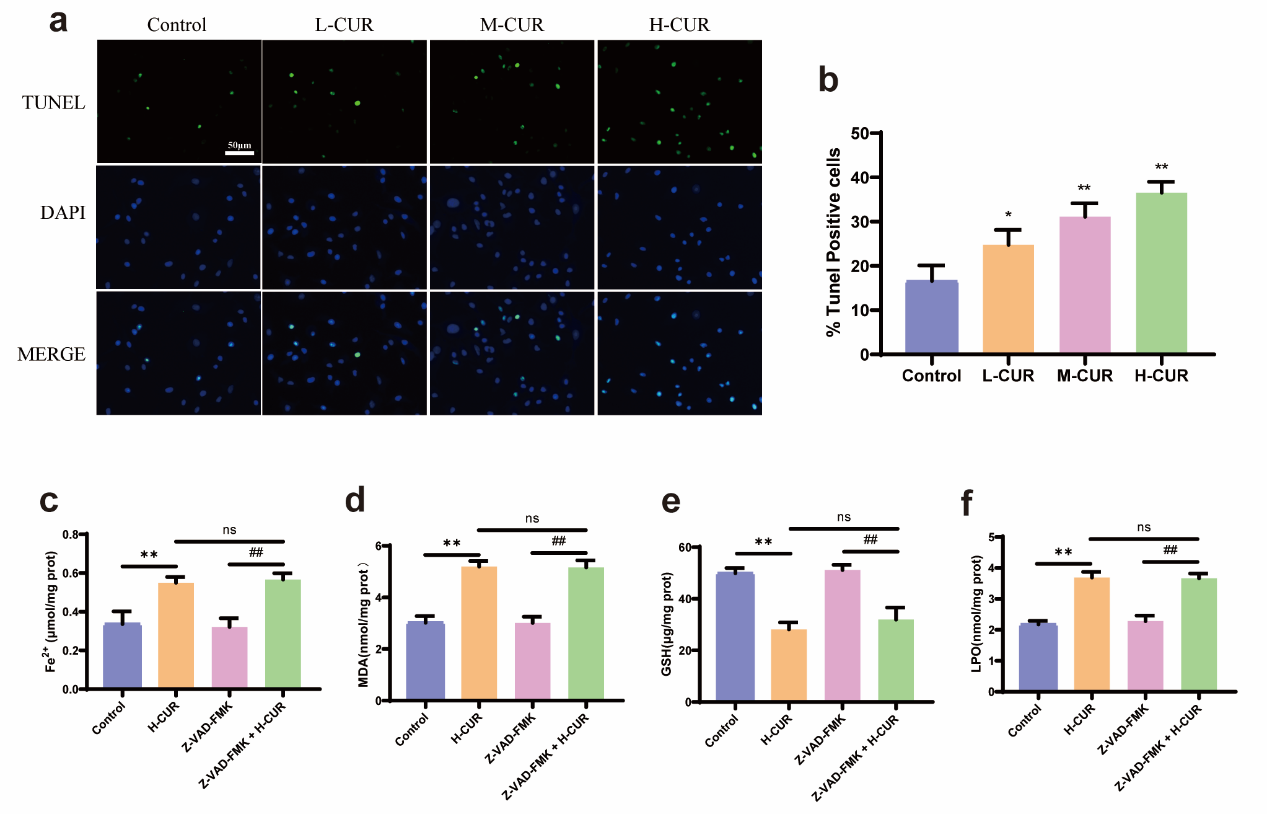


**Fig.S2**

**Fig. S2 Curdione-induced apoptosis does not affect the induction of ferroptosis.** a. SW480 cells were treated with 12.5 μM (*P*<0.5), 25 μM (*P*<0.01), and 50 μM (*P*<0.01) curdione for 48 h. TUNEL staining was used to detect apoptotic indices. DAPI staining was used as a reference for cell counts, and positive TUNEL staining showed the apoptosis of each group of cells. b. Percentage of apoptotic cells (green-labeled cells represent apoptosis). Values are expressed as the mean ± SD of three independent experiments. (n=3)^*^*P* < 0.05, ^**^*P* < 0.01 vs. control group. c-f. The kit assays the activity of Fe^2+^, MDA, GSH, and LPO in cells treated with 50 μM curdione and 1 μM Z-VAD-FMK (caspase inhibitor) for 1 h. Assays showed no significant change in the ferroptosis induced by curdione by apoptosis inhibitors. Values are expressed as the mean ± SD of three independent experiments. (n=3) ^**^*P* < 0.01, ^##^*P* < 0.01, ns, no significance.


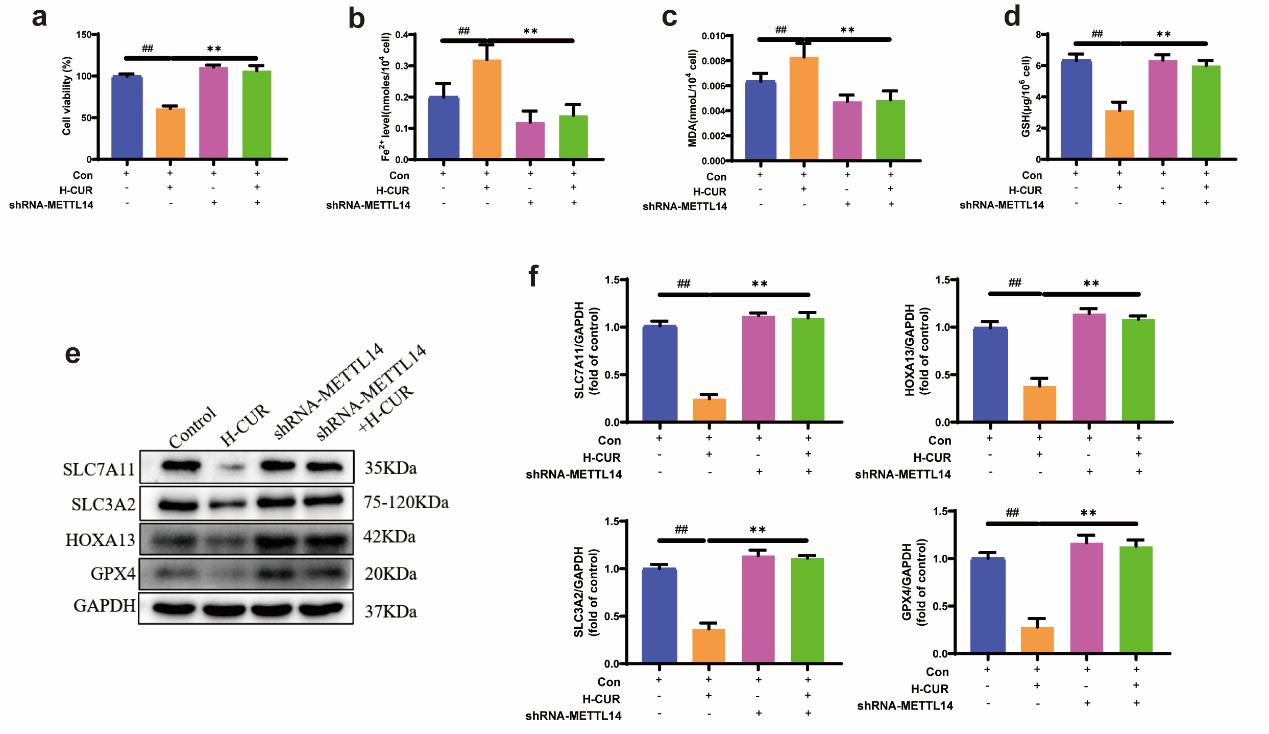


**Fig. S3**

**Fig. S3 Curdione promoted ferroptosis in colorectal cancer through the upregulation of METTL14.** The knockdown efficiency of METTL14 after 48 h of transfection of METTL14 shRNA SW480 cells was confirmed by Western blotting. Different concentrations (12.5 μM, 25 μM, and 50 μM) of curdione prepared from DMSO were added to CRC cells for 48 h. a. MTT assay for each group of transfected cell activities. ^##^*P* < 0.01 H-CUR group vs. control group. ^**^*P* < 0.01 H-CUR+shRNA-METTL14 group vs. H-CUR group. b-d. ELISA kits for the determination of Fe^2+^, MDA, and GSH in transfected cells. ^##^*P*<0.01 H-CUR group vs. control group. ^**^*P*<0.01 H-CUR+shRNA-METTL14 group vs. H-CUR group. h-i. The protein levels of SLC7A11, SLC3A2, HOXA13, and GPX4 in METTL14 knockdown CRC cells were detected by Western blotting. GAPDH was used as a control. Values are expressed as the mean ± SD of three independent experiments. (n=3) ^##^*P* < 0.01 H-CUR group vs. control group. ^**^*P* < 0.01 H-CUR+shRNA-METTL14 group vs. H-CUR group.
